# Homologous Recombination Deficiency Detection Algorithms: A Systematic Review

**DOI:** 10.3390/cancers15235633

**Published:** 2023-11-29

**Authors:** Lasse Ringsted Mark, Simone Karlsson Terp, Henrik Bygum Krarup, Mads Thomassen, Inge Søkilde Pedersen, Martin Bøgsted

**Affiliations:** 1 Department of Molecular Diagnostics, Aalborg University Hospital, DK-9000 Aalborg, Denmark; s.terp@rn.dk (S.K.T.); h.krarup@rn.dk (H.B.K.); isp@rn.dk (I.S.P.); 2 Department of Clinical Medicine, Aalborg University, DK-9000 Aalborg, Denmark; 3 Clinical Cancer Research Center, Aalborg University Hospital, DK-9000 Aalborg, Denmark; 4 Department of Clinical Genetics, Odense University Hospital, DK-5000 Odense C, Denmark; mads.thomassen@rsyd.dk; 5 Center for Clinical Data Science, Department of Clinical Medicine, Aalborg University and Research, Education, and Innovation, Aalborg University Hospital, DK-9000 Aalborg, Denmark; mboegsted@dcm.aau.dk

**Keywords:** homologous recombination deficiency, HRD, bioinformatics, cancer, algorithm

## Abstract

**Simple Summary:**

Homologous recombination deficiency (HRD) originates from genomic mutations or alterations in the homologous recombination repair pathway. Various promising tests have been developed to detect HRD. Some of these tests have shown good ability to predict response to Poly (ADP-ribose) polymerase inhibitors in cancer patients. However, a standardized way to define HRD has yet to be established. In this systematic review an overview of available HRD tests is provided. Important factors to consider are highlighted when planning clinical trials and studies involving HRD tests.

**Abstract:**

Homologous recombination deficiency (HRD) can arise from germline or somatic pathogenic variants as well as other genomic damage and epigenetic alterations in the HR repair pathway. Patients with tumors presenting with an HRD phenotype can show sensitivity to Poly (ADP-ribose) polymerase inhibitors (PARPis). Several promising tests to detect HRD have been developed based on different HRD definitions, biomarkers, and algorithms. However, no consensus on a gold standard HRD test has been established. In this systematic review, a comprehensive list of tests for the detection of HRD was identified and compared regarding HRD definition, biomarkers, and algorithms. PubMed’s Medline and Elsevier’s Embase were systematically searched, resulting in 27 eligible articles meeting the inclusion criteria. The primary challenge when comparing HRD tests lies in the lack of a consensus definition of HRD, as the HRD definition influences the proportion of samples being classified as HRD and impacts the classification performance. This systematic review provides an overview of available HRD tests that can inspire other researchers in searching for a gold standard HRD definition and highlights the importance of the factors that should be considered when choosing an HRD definition and tests for future planning of clinical trials and studies.

## 1. Background

Genomic profiling of tumors can be useful for understanding defects in DNA damage repair mechanisms and identifying patients who are candidates for targeted treatment [1,2,3,4]. Homolog recombination repair (HR) is a DNA damage repair mechanism that facilitates the repair of double-stranded breaks in DNA using a sister chromatid as template, thereby mediating an almost error-free repair of the double-stranded break [5]. Deficiency of the homologous recombination repair mechanism has been reported as a promoter of tumorigenesis as cells with HRD utilize more error-prone DNA repair mechanisms and accumulate mutations leading to genome instability [6,7,8,9]. HRD can be a result of germline or somatic pathogenic variants in genes involved in the HR repair pathway, primarily in the two key genes, *Breast cancer 1 (BRCA1)* and *Breast cancer 2 (BRCA2)* [10]. In addition, tumors can present with an HRD phenotype without identifiable germline or somatic HR variants. This HRD phenotype has yet to be fully characterized since HRD represents a broader phenomenon caused by abnormalities in the HR repair pathway, epigenetic alterations, or instability of the genome [11,12].

Patients with tumors presenting with an HRD phenotype show sensitivity to Poly (ADP-ribose) polymerase inhibitors (PARPis), which are targeted treatments inhibiting single-strand break repair, causing the phenomenon called synthetic lethality [2,3,4,13].

HRD-related genomic damage, often referred to as genomic scars, consists of different genomic aberrations which have been used in HRD tests as circumstantial evidence for HRD. The three most described genomic scars are loss of heterozygosity (LOH) [6], large-scale transition (LST) [7], and telomeric allelic imbalance (TAI) [8]. LOH is a genetic event where one of the alleles is missing [6], LST is chromosomal breaks between genomic regions [7], and TAI provides a measure for telomeric allelic imbalance [8]. Other measures of genomic scars providing an HRD phenotype are mutational signatures, originally described by Alexandrov et al. [14]. Mutational signatures are extracted by unsupervised clustering of point substitutions while considering adjacent sequence bases. *BRCA1* and *BRCA2* mutations have been described to be strongly associated with Signature 3 [14]. In addition, some tumors have shown a large contribution of Signature 3 without harboring *BRCA1* and *BRCA2* mutations, which might indicate that other genes with abnormalities might trigger similar mutational profiles [14].

Methylation of genes or pathogenic variants in genes in the HR repair pathway have also been used as biomarkers for HRD, as well as functional assays such as estimations of nuclear RAD51 foci [11,15,16,17,18].

Several promising tests to detect HRD have been developed based on different biomarkers and algorithms. Some HRD tests have been used in clinical trials to better define which cancers are most likely to have HRD. In the SOLO1 clinical trial, patients recently diagnosed with ovarian cancer showed benefits from PARPis harboring pathogenic BRCA variants [18]. The PRIMA and VELIA clinical trials have shown that ovarian cancer patients with HRD based on the HRD test myChoice from Myriad Genetics could benefit from a treatment combining platinum chemotherapy and PARPis [12,19]. However, one of the main challenges is the lack of consensus and a clear definition of HRD. This makes a direct comparison between HRD tests challenging as they are based on various definitions of HRD, biomarkers, and algorithms [11].

To our knowledge, a systematic review of tests for the detection of HRD has not yet been conducted. This systematic review assessed studies in which an HRD test was developed. The review was limited to HRD tests based on genomic/genetic data, including RNA profiling, but excluding HRD detection by functional assays and tests based solely on pathogenic variants, such as *BRCA1/2* variants. The aim of the review was to compare and evaluate the current HRD tests used for stratifying patients into HR groups while also addressing HRD definition and biomarkers used.

## 2. Materials and Methods

A systematic literature review was conducted following the Preferred Reported Items for Systematic Reviews and Meta-Analysis (PRISMA) guidelines [20].

### 2.1. Literature Search

PubMed’s Medline and Elsevier’s Embase databases were systematically searched for eligible articles. The full search strings for PubMed and Embase are presented in Supplementary Table S1. The search strings included three search groups with multiple search terms that represented the search group topic. The three search group topics were (1) homologous recombination deficiency, (2) HRD test, and (3) data type/method. The initial search was conducted on 13 October 2021, and a second search was conducted on 11 May 2022. The second search included seven search terms (see in Supplementary Table S1) identified as missing during the initial search as well as a relocation of a misplaced search term into its correct search group. Moving the misplaced search term into the correct search group did not add any relevant records compared to the initial search. Both searches were conducted with no limitation on the date of publication. Additional studies were identified by assessment of reviews and the bibliography of included articles. Two authors (LRM and SKT), independently and blinded to each other, screened titles and abstracts as well as full texts for assessment of eligibility using Covidence systematic review software (https://support.covidence.org/help/how-can-i-cite-covidence (accessed on 21 September 2022)) [21].

### 2.2. Inclusion and Exclusion Criteria

Studies were included in the review if fulfilling the following inclusion criteria: (1) concerning homologous recombination deficiency or BRCAness, (2) developing or training an algorithm/classifier for stratifying patients into HRD groups based on a threshold, (3) analyzing patient samples, (4) articles published in English, and (5) original research articles.

Articles were excluded if they used functional assays to stratify patients into HRD groups. Any discrepancies regarding article suitability were solved by consulting two other authors (MB and ISP).

### 2.3. Data Extraction

Two authors (LRM and SKT) critically reviewed included articles and independently extracted data manually into an Excel spreadsheet. Data concerning study type, disease, cohort size, sample material, HRD definition, algorithm description, and algorithm input were extracted. Studies were grouped according to their algorithm or classifier type. Key findings were also retrieved when available, including any available sensitivities, specificities, positive and negative predictive values (PPV and NPV), accuracies, or areas under the receiver operating characteristic (ROC) curve.

## 3. Results

### 3.1. Study Selection

**Figure 1:**
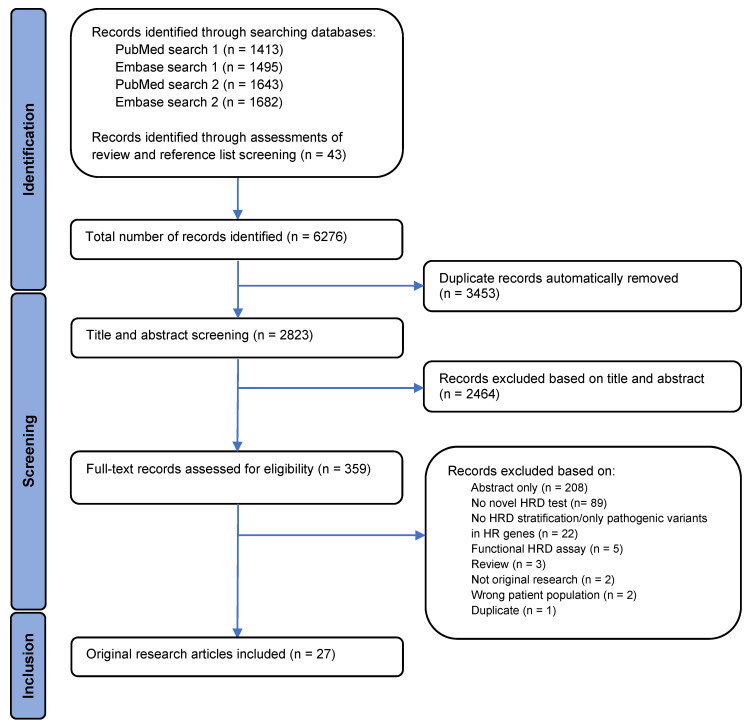
A PRISMA flowchart displaying the identification, screening, and inclusion process. The flowchart illustrates the filtration of identified records to the final number of articles included in the analysis, as well as exclusion reasons.

An overview of the study selection process is illustrated by a PRISMA flowchart in Figure 1. A total of 6276 records were identified, with 6233 records identified through searches in PubMed and Embase and 43 by assessment of reviews and the bibliography of the included articles. Automatic removal of 3453 duplicates was conducted using Covidence systematic review software, resulting in 2823 records left for the title and abstract screen. In the title and abstract screening, 2464 records were excluded, leaving 359 records available for full-text assessment of eligibility. Full-text screening of records resulted in 27 articles meeting the inclusion criteria.

The 332 full-text records which failed to meet the inclusion criteria were excluded for several reasons. Eighty-nine articles did not present a novel HRD test, and twenty-two records did not stratify patients into HR groups or only examined variants in HR-related genes. Five records used functional assays for HRD assessment, three records were identified as reviews, and two records were not original research. In addition, two records did not analyze patient samples and one record was identified as a duplicate that the automatic removal process in the Covidence systematic review software had not removed. The remaining records excluded were abstracts and, therefore, not eligible for full-text assessment.

### 3.2. Study Characteristics

The studies that are included in this review are based on different premises. These are things such as data origin, pre-analytical methods, and biological tissue types. This section will outline these various aspects. Table 1 displays the characteristics of the 27 included studies published between 1 January 2009 and 11 May 2022. Several types of cancers have been studied across the 27 studies, with the majority focusing on breast or ovarian cancer (Table 1).

Almost all studies included a training and a validation cohort, except for five studies which only included a training or analysis cohort. The number of validation cohorts included in each study ranged from one to four. The size of the training cohorts varied substantially from 21 to several thousand patients. Similar variation in cohort size was observed in the validation cohorts (for details see Table 1).

The studies included several cohorts, of which data accessibility differs, with publicly accessible cohorts and cohorts needing approved permission for access. Twelve studies included cohorts from The Cancer Genome Atlas (TCGA) database [46], three used METABRICS data [47], and nine used data from Gene Expression Omnibus (GEO) [48]. Five studies used data from the Nik-Zainal et al. [49] cohort that analyzed 560 breast cancer patients, and two used data from the PrECOG cohort [50]. In addition, some studies included internal cohorts, which are not directly available but only used and described in the given study (Table 1).

Data were obtained using different sample materials, and the analysis platforms differed substantially between the studies (Table 1). Fifteen studies included data obtained from arrays. Of these, nine studies included single-nucleotide polymorphism (SNP) arrays conducted on frozen tumor tissue. Four studies included a comparative genomic hybridization array (arrayCGH) conducted on formalin-fixed paraffin-embedded (FFPE) tumor tissue. A gene expression array was used by four studies conducted on frozen tumor tissue, with one study also using FFPE tumor tissue (Table 1). Five studies used microarrays without elaborating on the array type, with analyses conducted on frozen tumor tissue.

Nineteen studies used a next-generation sequencing (NGS) approach to obtain data for their algorithm input (Table 1). Six studies conducted whole-exome sequencing (WES) using frozen or FFPE tumor tissue. Whole-genome sequencing (WGS) was conducted by six studies, of which three used frozen tumor tissue and three used frozen and FFPE tumor tissue. Low coverage/shallow WGS was performed on frozen tumor tissue in two studies, with one of the studies also using FFPE tumor tissue. Seven studies used panel sequencing, of which five used FFPE tumor tissue and three used frozen tumor tissue. RNA sequencing (RNA-seq) was used in five studies, and microRNA sequencing (miRseq) was performed in one study, with all studies using frozen tumor tissue and two studies also using FFPE (Table 1)

Other platforms were also used for obtaining data, with one study using digital multiplex ligation-dependent probe amplification (digitalMLPA) on FFPE tumor tissue and another using MLPA on FFPE and frozen tumor tissue (Table 1).

### 3.3. Definition of HRD

**Figure 2:**
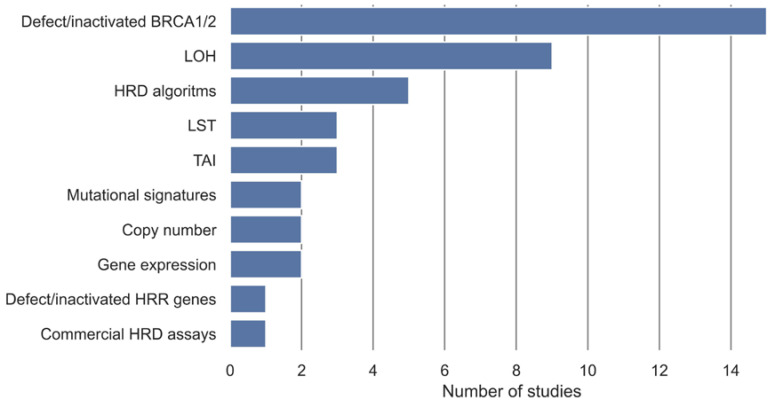
Overview of gold standards for definition of HRD, either alone or in combination, and their frequencies in the included studies. Defect/inactivated BRCA1/2 covers biallelic or monoallelic methylation, somatic, and germline variants in BRCA1 and BRCA2. Defect/inactivated homologous recombination repair (HRR) genes cover biallelic or monoallelic methylation, somatic, and germline variants in genes involved in the homologous recombination repair pathway in addition to BRCA1 and BRCA2. Loss of heterozygosity (LOH) covers measures of LOH, ranging from LOH in individual genes to genome wide LOH. HRD algorithms cover HRD defined from an HRD algorithm developed in another study.

In the included studies, different measures, either alone or in combination, have been used to define a gold standard for samples being either non-HRD or HRD (Table 2). These gold standards are used as class labels in the development of the HRD tests. These measures have been categorized into ten categories (Figure 2). Methylation, somatic, and germline variants in *BRCA1* and *BRCA2* were the most used gold standard measures of HRD and have been used in 15 studies. Nine studies used a measure of LOH as the definition of HRD, and five used an already-established HRD algorithm as the gold standard. LST and TAI were used as the gold standard in three studies, and a commercial HRD assay was used in one study. Gene expression, copy number, and mutational signatures were each used in two studies as the gold standard for HRD, and methylation, somatic, or germline variants in HR genes were used in one study.

### 3.4. HRD Detection Algorithms

**Figure 3:**
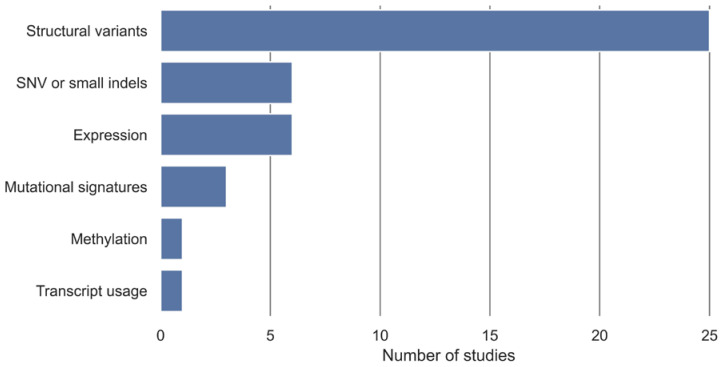
Overview of algorithmic input and number of studies that are included. Structural variants include measures such as copy number, loss of heterozygosity (LOH), large-scale transitions (LST), ploidy, percentage of genomic LOH, large-scale genomic alterations (LGA), structural variants, and methylation copy number. Expression includes gene expression and miRNA expression. SNV and small indels include point mutations, single-base substitution, and smaller indels. Some HRD tests have been based on more than one algorithm input.

In 16 studies, the development of HRD tests has been based on existing classification algorithms for classifying samples into non-HRD and HRD groups with an HRD definition used as a class label. The additional 11 studies have developed a novel classification algorithm to classify samples into non-HRD and HRD groups (Table 2). The algorithm input data have been stratified into categories, displayed in Figure 3, with structural variants being the most utilized algorithm input, followed by SNV or small indels and expression.

Two studies used a nearest centroid classifier for discriminating non-HRD and HRD groups, with Lips et al. 2011 [23] reporting the *BRCA1-like MLPA classifier* to classify BRCA1-like breast cancers based on copy number input. Severson et al. 2017 [29] presented the *BRCA1ness signature* of breast cancer based on gene expression. The nearest centroid classifier finds the centroid for all predictors per class, being the mean value of each predictor, and new samples are then assigned a class based on the closest centroid [51].

Four studies based their HRD test on a shrunken centroid model, which is based on the same concept as a nearest centroid but includes an additional step, shifting class-based centroids towards the centroid of all features. If a feature is shrunken down to the centroid of all features, it can be excluded as the feature does not add any discriminating information. This additional step in the shrunken centroid model acts as a feature selection for the model [52]. Of the four studies using a shrunken centroid model, Joosse et al. 2009 [22] reported the *BRCA1 classifier*, which was developed to classify BRCA1-like breast cancers. In the study by Joosse et al. 2012 [24], a similar classifier was developed, the *BRCA2 classifier*, for the classification of BRCA2-like breast cancers. Lips et al. 2020 extended the two algorithms from Joosse et al. [22,24] to a new platform and presented the *BRCA1-like digitalMLPA classifier* and *BRCA2-like digitalMLPA classifier* also based on copy number. Schouten et al. [40] applied the same methodological approach as Joosse et al. [22,24] to develop two HRD tests that were specific for ovarian cancer, the *Ovarian cancer BRCA1-like classifier* and *Ovarian cancer BRCA2-like classifier*, which both are based on copy numbers as input.

Chen et al. [33] reported the *BRCA1-like classifier* that was developed to classify BRCA1-like breast cancers based on a support vector machine (SVM) classifier using copy number as input. An SVM classifier finds a hyperplane in the feature space, which can be used to separate the classes of data points. The optimum hyperplane is the plane with the maximum margin between points from separate classes. The classifier can then be applied to new samples and assign a class label [53].

Leibowitz et al. [43] reported the *HRD-RNA* for pan-cancer using a logistic regression model. Logistic regression models the probability of classifying a sample into possible outcomes with a number of dependent variables [51]. Leibowitz et al. [43] also included the *HRD-DNA* based on genome-wide LOH.

Four studies based their HRD test on a least absolute shrinkage and selection operator (LASSO) regression model, a regression analysis used as a technique to reduce model complexity. LASSO selects and shrinks the model to use the optimum number of features based on regularization [54]. Of the four studies using LASSO, Zhuang et al. [41] reported the *24 gene pair* (*24-GPS)* classifier that provided an HRD signature for pancreatic cancer based on gene expression input. Davies et al. [28] developed *HRDetect* to classify *BRCA1*/*BRCA2*-deficient breast, ovarian, and pancreatic cancer based on a LASSO logistic regression model that included mutational signatures, LOH, and indels from WGS as input. Diossy et al. [31] extended the HRDetect from Davies et al. [28] to use mutational signatures, LOH, and indels from WES data of breast cancer and brain metastases. They presented the retrained *WES-HRDetect* based on a LASSO logistic regression model. The study by Liao et al. [44] also used a LASSO logistic regression model for their *Transcriptomic HRD score* for breast cancer, which was based on gene expression input.

Three studies used a random forest (RF) model, which is an ensemble method constructed by a large number of independently trained decision trees where features for each decision tree are selected randomly [55]. Of the three studies using RF-based models, Nguyen et al. [37] reported the pan-cancer *Classifier of HOmologous Recombination Deficiency (CHORD)I* based on single-base substitutions, indels, and structural variants. Barenboim et al. [38] reported the *DNA-methylation-based RF classifier* providing a BRCAness signature for osteosarcoma using methylation copy numbers from array data. Kang et al. [42] reported *transcriptional HRD (tHRD)* based on transcript usage.

Gulhan et al. [34] developed *Signature Multivariate Analysis (SigMA)*, which uses an approach based on mutational signatures extracted by non-negative matrix factorization (NMF). NMF is an unsupervised machine learning algorithm that factorizes the original dataset into a feature set and a coefficient set. Each feature set has an associated weight in the coefficient set. The feature and coefficient set can be used to select, reduce, or analyze individual features of data [56].

Most of the 11 studies with a novel classification algorithm to classify samples into non-HRD and HRD groups were based on genomic scar measures.

Popova et al. [7] reported *LST* as a classifier of HRD in breast cancer. The LST test consists of a two-step decision rule with segregation of tumors based on ploidy followed by segregation according to the number of LST counts. *Large-scale genomic alterations (LGA)*, which are reported to correspond to LST, were used by Eeckhoutte et al. [35], who developed *ShallowHRD* for breast and ovarian cancer based on the sum of LGA counts from WGS at low coverage (~1X coverage).

Abkevich et al. [6] reported *HRD-LOH* as a classifier of HRD in ovarian cancer based on the sum of LOH segment counts. Smyth et al. [32] also included a measure of LOH, *the genomic LOH*, for HRD classification in esophagogastric cancer. The genomic LOH test was based on the sum of the lengths of included LOH segments divided by the length of the interrogated genome.

Telli et al. [16] reported the *combined homologous recombination deficiency score (HRD score)* for breast and ovarian cancer, which consists of the numeric sum of LOH, LST, and TAI counts. Similar to the HRD score, Chen et al. [39] reported a *genomic scar algorithm (GSA)* to provide a measure of HRD for breast and ovarian cancer. The GSA consists of the numeric sum of LST, TAI, and LOH, which then is subtracted by a correction coefficient multiplied by a ploidy value.

The study by Watkins et al. [27] reported *scores of chromosomal instability scarring (SCINS)* for breast and ovarian cancer, consisting of four scores based on different types of allele-specific copy-number profiles.

Zhang et al. [26] developed a *genomic instability score* for ovarian cancer based on the sum of the number of copy number changes and somatic mutations multiplied by a constant.

Tandem duplications as a genomic scar that provided a measure of HRD in *BRCA1*-type breast cancers were reported by Qu et al. [45], who developed the *tandem duplications score (TD-score)* based on the counts of small (<10 kb) tandem duplications.

Two studies based their HRD test on other measurements of HRD than genomic scars, with Lu et al. [25] reporting the *hypothesized HR-deficiency score (HRDS)* classifying breast and ovarian cancer based on gene expression levels and Wang et al. [30] developing the *10-miRNA-score* for HRD prediction of ovarian cancer based on the expression levels of miRNA.

### 3.5. HRD Test Performance

**Figure 4:**
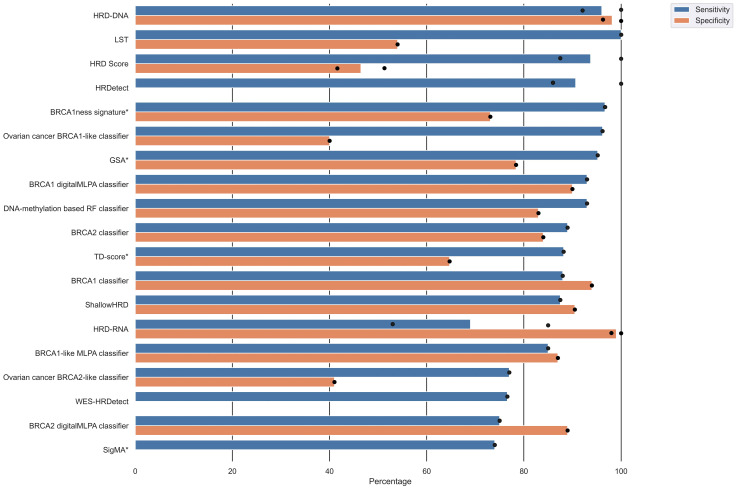
Overview of sensitivity and specificity of the HRD tests. Several studies reported multiple validation results. Dots represents individual reported validation results, and bars represents mean sensitivity/specificity. Note that not all studies reported both sensitivity and specificity. Algorithms marked with an asterisk (*) next to the algorithm alias have only been internally validated.

The included studies provided a variety of performance measures and validation results (Table 2) based on a study-individual gold standard of HRD. Sixteen studies reported the sensitivity of their HRD test, of which five studies reported more than one sensitivity due to multiple validations. Corresponding specificities were only reported in 13 studies, of which four provided more than one specificity. An overview of the sensitivity and specificity of the different HRD tests is displayed in Figure 4 and Table 2. The sensitivity ranged from 53% to 100%, and the specificity ranged from 40% to 100%. The HRD test with the highest sensitivity and corresponding specificity was HRD-DNA [43], followed by BRCA1-like digitalMLPA classifier [36], BRCA1 classifier [22], ShallowHRD [35], BRCA1-like MLPA classifier [23], DNA-methylation-based RF classifier [38], and BRCA2 classifier [24]. The HRD score [16], LST [7], BRCA1ness signature [29], and Ovarian cancer BRCA1-like classifier [40] had high sensitivity, but the corresponding specificities were relatively low (Figure 4). HRDetect [28] also had a high sensitivity but did not report a corresponding specificity.

Nine studies provided an area under the curve (AUC) ranging from 75% to 100% (Table 2), with AUC being highest for HRD-DNA [43], HRD-RNA [43], CHORD [37], 24-GPS [41], and WES-HRDetect [31].

The accuracy was reported in five studies ranging from 72 to 91% (Table 2), with accuracy being highest for the BRCA1-like digitalMLPA classifier [36].

## 4. Discussion

This systematic literature review identified 27 studies in which an HRD test was developed or trained to stratify patients into HR groups, with all HRD tests being based on genomic or RNA profiling.

In the included studies, the definition of HRD was rather heterogeneous and lacked consensus between the studies. The definition of HRD was based on multiple measures used either alone or in combination (Figure 2), with defect *BRCA1/2* and LOH being the most frequently used measures of HRD.

Most of the HRD tests included in this review were developed to predict HRD in breast and ovarian cancer, followed by prostate and pancreatic cancers (Table 1). The rationale for developing HRD tests for these cancer types could be that more than 15% of breast, ovarian, and pancreatic cancers and 14% of prostate cancer have mutations in HR-related genes [57]. In addition, mutations in *BRCA1/2* are associated with an increased lifetime risk of developing breast, ovarian, prostate, and pancreatic cancers [58]. Furthermore, early studies of PARPis showed promising results in *BRCA1/2*-deficient cells, which built the foundation for clinical trials investigating PARPi response in ovarian cancer. Later, clinical trials with PARPis were expanded to breast, prostate, and pancreatic cancers [59]. As the majority of HRD tests included in this review are developed for HRD detection in ovarian and breast cancer, it is important to recognize that other cancer types might include different HRD patterns. For instance, Diossy et al. [31] found that brain metastases from breast cancer tend to have a higher HRD score than primary breast cancer, which should be considered in a clinical context. The studies included in this review have used a variety of different tissue types in the development of the HRD test. It is important to recognize that HRD measures generated from various tissue types might produce different results and should be validated accordingly. Furthermore, there might be several important considerations when implementing an HRD test in clinical practice, such as the stability of the material used, cost of running the analysis, and the turnaround time.

The lack of consensus on the HRD definition and a gold standard measure of HRD provides a growing problem when developing HRD tests. This became evident during the PRIMA and VELIA clinical trials, where patients with HRD-positive tumors, defined by the myChoice HRD test based on LOH, LST, and TAI, responded to combination treatment with PARPis. However, the response to PARPis was not at the same magnitude as for patients with HRD defined by somatic or germline pathogenic variants in *BRCA1/2* [12,19]. This highlights that defects in *BRCA1/2* are one of the most robust measures of HRD, although it does not cover all phenotypes of HRD. In addition, secondary or reversion mutations in *BRCA1/2* have been found to restore the functionality of the HR mechanism [60,61]. Hence, genomic scars, such as LOH, LST, and TAI, provide an imperfect measure of the HR function, as these measures are results of prior HRD exposure [62,63]. HRD tests based on functional assays can assess the HR mechanism’s functionality, potentially providing a more precise and clinically relevant measure of HRD. Unfortunately, such functional assays are in the early stages of development and are prone to a similar lack of consensus on HRD definition as other HRD tests, making clinical implementation difficult [64].

Most HRD tests were trained with HRD defined as various measures specific to *BRCA1* and/or *BRCA2*. There is, however, evidence that HRD can arise based on variants in a wider set of genes related to the HR pathway [10,11]. Interestingly, only ShallowHRD was developed based on an HRD definition, including more HR-related genes than *BRCA1* and *BRCA2* [35]. HRD definitions mainly based on variants in *BRCA1/2* or genes related to the HR pathway have been referred to be the etiology or origin of HRD, as these variants are the main reason that a given gene is inactivated or defective [11]. HRD tests such as tHRD, SigMA, SCINS, WES-HRDetect, genomic LOH, DNA-methylation-based RF classifier, and transcriptomic HRD score defined HRD as various measures of structural variants across the genome, which have been referred to as genomic scars or prior HRD exposure. HRD tests based on genomic scars aim to detect a genomic pattern resulting from prior HRD exposure without detecting the underlying reason [11].

The performance of the included HRD tests varied substantially (Figure 4 and Table 2), with the HRD tests HRD-DNA [43], BRCA1 digitalMLPA classifier [36], and BRCA1 classifier [22] having average sensitivities and specificities above 90%. In common for these HRD tests is the use of copy number as an algorithmic input and a definition of HRD as biallelic loss or variants in *BRCA1* and/or *BRCA2*. The BRCA1 digitalMLPA classifier, however, indirectly defines HRD as variants in *BRCA1* by using the BRCA1 classifier as the HRD definition, which makes interpreting this algorithm somewhat difficult. Although these HRD tests perform well when predicting samples with biallelic loss or variants in *BRCA1* and/or *BRCA2*, their utility might be limited by their HRD definition.

Some studies evaluated the HRD tests’ performance by AUC, with HRD-DNA, HRD-RNA, CHORD, 24-GPS, and WES-HRDetect all having AUCs above 96%. However, the 24-GPS was not evaluated in an external dataset, which is why this test needs further evaluation to validate the performance. Interestingly, for these five HRD tests, their individual input biomarkers were also included in their individual HRD class label definition, contrary to most of the other studies using various biomarkers to predict an HRD class label defined as, for instance, *BRCA* mutations.

Prediction models are usually validated using sensitivity and specificity [65]. A high sensitivity describes a model’s ability to predict true positives, and specificity describes the model’s ability to predict true negatives. Hence, a model having both high sensitivity and specificity minimizes false positives and false negatives [65]. However, many of the included studies used a non-classical approach to assess performance in which they suggested that false-positive samples, impacting the specificity, might not be misclassified samples but instead samples which harbor similar patterns as HRD-positive samples. Thereby, the false-positive samples are suggested to be true HRD samples that do not comprise the measures used as the HRD definition. This complicates the comparison of performance for the HRD tests even further. For instance, Davies et al. [28] found that one-third of tumors with a high HRDetect score could not be verified as *BRCA* mutated, but they argued that these tumors seem biologically comparable to *BRCA*-mutated tumors and might respond similar to PARP inhibitors.

A way to empirically compare the performance of the HRD tests could be based on drugs targeting HRD, such as PARPis and platinum chemotherapy, with drug response being used as a surrogate marker for HRD. Although the response to PARPis might be affected by other mechanisms, the approach could be useful for the comparison of various HRD tests and support the clinical utility of the tests [11,64].

In a clinical context, it is important to be aware of the HRD definition and how it influences the proportion of patients selected as HRD-positive. For instance, in the VELIA clinical trial, the percentage of patients eligible for PARPi treatment when considering HRD as *BRCA* mutations were 26% compared to 55% when considering HRD as either *BRCA* mutations or a measure of genomic scars defined by the myChoice HRD test [19]. This further highlights the importance of the HRD definition as it can highly influence the proportion of patients with HRD-positive tumors and, thereby, patients eligible for treatment. When summarizing the issues concerning HRD definition and the non-classical approach to specificity, it is intuitive to believe that there will be some potential limitations in identifying the group of patients who have functional HR repair and, thereby, likely not to benefit from treatments targeting HRD. This issue has been raised elsewhere in several studies [11,64].

The main limitation of this review was the limited opportunity to compare HRD tests based on their performance measures, as many HRD tests lacked information regarding performance and/or did not conduct an external validation of the HRD test, as well as the lack of consensus of HRD definitions. Furthermore, the studies were reported in a rather heterogeneous manner, which hindered a clear interpretation of the effects of, e.g., analysis platform, data input, or disease on the performance of the HRD tests. However, this systematic review provides a detailed summary of the numerous parameters included in the HRD detection algorithms and addresses the challenges of choosing a suitable HRD test due to the heterogeneity of the parameters. Although we conducted the systematic literature search using two widely used databases and assessed reviews of relevant topics and the bibliographies of the included articles, we cannot exclude having missed relevant articles. Studies published in languages other than English and those without available full text were not included in the review, so we cannot exclude a publication bias. In addition, the review was limited to including HRD tests based on genomic and RNA profiling and excluding HRD detection by functional assays and HRD tests based solely on HR-related pathogenic variants. The decision to exclude HRD tests based on functional assays and studies based solely on HR-related pathogenic variants was based on a large number of studies eligible for full-text review when including these HRD tests too. Furthermore, we limited inclusion to studies that developed or trained a novel HRD test. Studies that evaluated an HRD test in additional cohorts without training or modifying the HRD test were excluded due to the significant addition of studies eligible for full-text screening. Therefore, we cannot exclude that this limitation on the HRD tests included can bias our evaluation of the HRD tests’ performance.

## 5. Conclusions

This systematic review provided an overview of the HRD tests that have been developed and summarized the variety of different biomarkers, algorithms, and HRD definitions used. The primary challenge for the comparison of HRD tests lies in the definition of HRD. The performance of the included HRD test varied with some performing better than others. However, this review also highlights that the HRD definition influences the proportion of samples classified as HRD and impacts the classification performance.

With this systematic review comparing HRD tests, we have provided an overview that can inspire other researchers in searching for a gold standard HRD definition, as this field requires one such to most suitably classify tumors as HRD or non-HRD. In addition, we have highlighted the importance of the factors that should be considered when choosing an HRD definition and HRD test for future planning of clinical trials and studies, as a consensus definition of HRD is truly needed.

## Figures and Tables

**Table 1:** Characteristics of the eligible studies, including cancer type, cohort size, tissue sample type, methods, and a description of the developed algorithms.

Author (et al.)	Year	Algorithm	Cancer Type	Cohort	Cohort Size	Tumor Tissue Type	Method	Algorithm Description
Joosse [22]	2009	BRCA1 classifier	Breast	gBRCA1 mutated	34 T	FFPE	Array-CGH	Shrunken centroid model
				Sporadic	48 T			
				HBOC	48 V			
Lips [23]	2011	BRCA1-like MLPA classifier	Breast	NKI-clinical genetics series	34 T 18 V	FFPE	MLPA	Nearest shrunken centroid model
				NKI-AVL neoadjuvant chemotherapy	50 T 8 V	Frozen		
				Randomized trial series	46 V	FFPE		
				Deventer series	69 A	FFPE		
Abkevich [6]	2012	HRD-LOH	Ovarian	Gynecology Cancer Banks at MDACC and UCSF	152 T	Frozen	SNP array	Sum of LOH segment counts
				Magee-Womens Hospital of UPMC	53 V			
				TCGA ovarian cancer	435 V			
Joosse [24]	2012	BRCA2 classifier	Breast	gBRCA2 mutated	28 T 19 V	FFPE	Array-CGH	Shrunken centroid model
				Sporadic	28 T 19 V			
				HBOC	89 V			
				gBRCA1 mutated (Joosse et al. 2009)	34 A			
Popova [7]	2012	LST	Breast	BLC	80 T 60 V	Frozen	SNP array	Two-step decision rule. First, segregate tumors based on ploidy and second, segregate according to number of LST counts.
Lu [25]	2014	Hypothesized HR-deficiency score (HRDS)	Breast Ovarian	TCGA ovarian cancer	167 T 141 V	Frozen	WES	Score based on gene expression levels
				TCGA breast cancer	127 A	Frozen		
				Bonome dataset	185 A	Frozen		
				Yoshihar dataset	300 A	Frozen		
				Tothill dataset	285 A	Frozen		
Zhang [26]	2014	Genomic instability score	Ovarian	TCGA ovarian cancer	325 T	Frozen	NGS panel SNP array	Score based on CNC regions and somatic mutations
Watkins [27]	2015	Scores of chromosomal instability scarring (SCINS)	Breast Ovarian	Guy’s Hospital King’s College London TNBC	142 A	Frozen	SNP array Gene expression microarray	Four scores based on different types of allele-specific copy-number profiles
				METABRIC TNBC	115 A	Frozen		
				TCGA TNBC	80 A	Frozen		
				PrECOG TNBC	80 A	Frozen		
				TCGA HGSC	299 A	Frozen		
Telli [16]	2016	Combined homologous recombination deficiency score (HRD score)	Breast Ovarian	Breast cancer: TCGA Timms et al. 2014 cohort	497 T	Frozen	Microarray SNP array WES Capture panel NGS	Numeric sum of LOH, LST, and TAI counts
				Ovarian cancer: TCGA Hennesy et al. 2010	561 T	Frozen		
				Breast cancer: PrECOG 0105	93 A	FFPE Frozen		
				Breast cancer: Neoadjuvant cisplatin trials	79 A	FFPE Frozen		
Davies [28]	2017	HRDetect	Breast Ovarian Pancreatic	Nik-Zainal et al. 2016 cohort	560 T	Frozen	WGS	LASSO logistic regression model
				Low coverage simulated Nik-Zainal et al. 2016 cohort	560 V	N/A		
				Breast cancer	80 V	N/A		
				Pancreatic cancer	96 V	Frozen		
				Breast cancer	3 V	FFPE		
				Ovarian cancer	73 V	Frozen		
				TNBC	9 A	Needle biopsy		
Severson [29]	2017	*BRCA1*ness signature	Breast	RATHER cohort	128 T	Frozen	Array	Nearest centroid model
				I-SPY 2 trial	116 V			
Wang [30]	2017	10-miRNA-score	Ovarian	TCGA ovarian cancer	319 A	Frozen	miRNA microarray miRNA-Seq	Score based on miRNA expression levels
				TCGA ovarian cancer samples	136 A		miRNA-Seq	
				TCGA breast cancer	657 A		miRSeq	
Diossy [31]	2018	WES-HRDetect	Breast Brain metastases	Matched primary breast cancer and brain metastasis	21 T	FFPE Frozen	WES	LASSO logistic regression model
					17 V	FFPE		
Smyth [32]	2018	Genomic LOH	Esophagogastric	REAL3 cohort	158 T	FFPE	NGS panel	Sum of the lengths of included LOH segments divided by the length of the interrogated genome.
Chen [33]	2019	BRCA1-like classifier	Breast	GSE9021 GSE9114	74 T	FFPE	Array-CGH	Support vector machine
				GSE18626	106 V	FFPE		
				TCGA breast cancer	957 A	Frozen		
				METABRIC breast	1968 A	Frozen		
Gulhan [34]	2019	Signature Multivariate Analysis (SigMA)	Breast Osteosarcoma Ovarian Pancreatic Prostate	TCGA Breast cancer	730 T	Frozen	WGS	Likelihood-based measure combined with clustering using non-negative matrix factorization
				Down-sampled TCGA breast cancer	730 T	Simulated	Down-sampled WGS	
				Breast cancer (MSK-IMPACT data)	878 V	FFPE	Capture panel NGS	
				Nik-Zainal et al. 2016 cohort	560 V	Frozen	WGS	
Eeckhoutte [35]	2020	ShallowHRD	Breast Ovarian	Primary breast and ovarian cancer	26 T	Frozen	Shallow WGS	Sum of LGA counts
				Primary breast and ovarian cancer	4 T	FFPE	Shallow WGS	
				Patient-derived xenografts	39 T	Frozen	Shallow WGS	
				TCGA-BRCA	108 normal T 79 tumor V	N/A	Down-sampled WGS	
Lips [36]	2020	BRCA1-like digitalMLPA classifier BRCA2-like digitalMLPA classifier	Breast	Cohort for BRCA1-like digitalMLPA classifier	71 T 70 V	FFPE Frozen	digitalMLPA	Shrunken centroid model
				Cohort for BRCA2-like digitalMLPA classifier	55 T 56 V			
				The Dutch high-dose trial	122 A			
Nguyen [37]	2020	Classifier of HOmologous Recombination Deficiency (CHORD)	Pan-cancer	Metastatic Pan-cancer (HMF Priestley)	3824 T	Frozen	WGS	Random-forest-based model
				Primary pan-cancer (PCAWG)	1854 V			
				Nik-Zainal et al. 2016 cohort	560 V			
Barenboim [38]	2021	DNA-methylation-based RF classifier	Osteosarcoma	Osteosarcoma	43 T 20 V	Frozen	RNA-seq	Random forest model
Chen [39]	2021	Genomic scar algorithm (GSA)	Breast Ovarian	Breast and ovarian cancer	195 T	FFPE	MGI panel sequencing	Numeric sum of LST, TAI, LOH subtracted by correction coefficient multiplied a ploidy value
Schouten [40]	2021	Ovarian cancer BRCA1-like classifier Ovarian cancer BRCA2-like classifier	Ovarian	NKI and EMI cohort	73 T	FFPE	Array-CGH	Shrunken centroids classifier
				AGO-TR1	523 A	FFPE blood	Low-coverage WGS	
Zhuang [41]	2021	24 gene pairs (24-GPS)	Pancreatic	TCGA	147 T	Frozen, blood	RNA-seq	LASSO regression model
				ICGC-AU	95 V	N/A	Gene expression array	
				GSE17891	27 V	FFPE		
				GSE57495	63 V	Frozen		
Kang [42]	2022	Transcriptional HRD (tHRD)	Breast Ovarian	TCGA-BRCA	272 T	Frozen	RNA-seq WGS WES	Random-forest- based model
					116 V			
				TCGA-OV	130 T			
					32 V			
				NAC	27 A	Frozen FFPE		
				PR	36 A			
				OM	24 A			
				OS	33 A			
Leibowitz [43]	2022	HRD-DNA	Pan-cancer	Breast cancer	483 T	FFPE Blood	NGS panel	gwLOH
					64 V			
					1511 A			
				Ovarian cancer	289 T			
					69 V			
					858 A			
		HRD-RNA		Pancreatic cancer	1375 T		RNA-seq panel	Logistic regression model
					301 D			
					165 V			
					1927 A			
				Prostate cancer	925 T			
					204 D			
					119 V			
					1536 A			
				Other	9921 T			
					2125 D			
					1113 V			
					20772 A			
Liao [44]	2022	Transcriptomic HRD score	Breast	TCGA	1084 T	Frozen	WES Gene expression array	LASSO logistic regression model
				GSE25055	114 A	Fine-needle aspiration core biopsy	Gene expression array	
				GSE25065	64 A	Fine-needle aspiration core biopsy	Gene expression array	
				GSE41998	140 A	Frozen	Gene expression array	
				METABRIC	299 A	Frozen	Gene expression array	
				Nik-Zainal et al. 2016 cohort	75 V	Frozen	WGS Gene expression array	
Qu [45]	2022	Tandem duplications score (TD-score)	Breast	Nik-Zainal et al. 2016 cohort	266 T	Frozen	RNA-seq WGS	Score of TD counts

Abbreviations: A: Analysis cohort; BLC: Basal-like breast carcinomas; BRCA1: Breast cancer 1; BRCA2: Breast cancer 2; CGH: Comparative genomic hybridization; CHORD: Classifier of HOmologous Recombination Deficiency; CNC: Copy number counts; D: Discovery cohort; FFPE: Formalin fixed Paraffin Embedded; GPS: Gene pairs (24-GPS); GSA: Genomic Scar algorithm; gwLOH: Genome wide LOH; HGSC: High-grade serous carcinoma; HRD: Homologous recombination deficiency; HRDS: Hypothesized HR-deficiency score; LASSO: Least absolute shrinkage and selection operator; LGA: Large-scale genomic alterations; LOH: Loss of heterozygosity; LST: Large-scale transitions; MIP: Molecular Inversion Probe; miRNA: MicroRNA; MLPA: Multiplex ligation-dependent probe amplification; mRNA: Messenger RNA; N/A: Not available; NGS: Next generation sequencing; RF: Random Forest; SCINS: Scores of chromosomal instability scarring; Seq: Sequencing; SigMA: Signature Multivariate Analysis; SNP: Single nucleotide polymorphism; T: Training cohort; TAI: Telomeric allelic imbalance; TCGA: The Cancer Genome Atlas; TD: Tandem duplications; tHRD: Transcriptional HRD; TNBC: Triple negative breast cancer; V: Validation cohort; WES: Whole exome sequencing; WGS: Whole genome sequencing.

**Table 2:** An overview of algorithm input, study type, performance measures, and HRD gold standard. Internal validation is defined as validation primarily conducted on training data. This also includes cross-validation if no external data have been used. External validation is defined as validation conducted on external data or if a part of the dataset has been held out from training with the main purpose to use as a validation set.

Author (et al.)	Algorithm	Algorithm Input	Study Type ^a^	Validation	Performance	Gold Standard of HRD
Joosse [22]	BRCA1 classifier	Copy number	Predictive	External	Sensitivity: 88% Specificity: 94% PPV: 93% NPV: 88%	*BRCA1* germline variants
Lips [23]	BRCA1-like MLPA classifier	Copy number	Predictive Explanatory	External	Sensitivity: 85% Specificity: 87% Accuracy: 86%	Algorithm developed by Joosse et al. [22]
Abkevich [6]	HRD-LOH	LOH	Explanatory	No validation	N/A	*BRCA1/2* methylation, germline, and somatic variants LOH *BRCA1* expression
Joosse [24]	BRCA2 classifier	Copy number	Predictive	External	Sensitivity: 89% Specificity: 84% PPV: 85% NPV: 89%	*BRCA2* germline variants
Popova [7]	LST	LST Ploidy	Predictive	External	Validation: Sensitivity: 100% Specificity: 54%	*BRCA1/2* germline and somatic variants *BRCA1* promoter methylation
Lu [25]	HRDS	Gene expression	Descriptive Explanatory	No validation	N/A	*BRCA1/2* variants
Zhang [26]	Genomic instability score	Copy number Point mutation Indels	Explanatory	No validation	N/A	*BRCA1/2* variants *BRCA1* methylation
Watkins [27]	SCINS	Copy number	Descriptive Explanatory	No validation	N/A	Copy number measure
Telli [16]	HRD score	LOH LST TAI	Explanatory	External	PrECOG 0105: Sensitivity: 100% ^a^ Specificity: 41.6% ^a^ Neoadjuvant cisplatin trials cohort: Sensitivity: 87.5% ^a^ Specificity: 51.3% ^a^	*BRCA1/2* variants LOH *BRCA1* methylation
Davies [28]	HRDetect	Mutational signatures LOH Indels	Predictive	External	Breast cancer cohort: Sensitivity: 86% Low-coverage WGS breast cancer cohort: Sensitivity 86% Ovarian and pancreatic cancer cohort: Sensitivity: approaching 100%	*BRCA1/2* variants
Severson [29]	BRCA1ness signature	Gene expression	Predictive Explanatory	Internal	Sensitivity: 96.7% (T) Specificity: 73.1% (T)	Algorithm developed by Lips et al. [23].
Wang [30]	10-miRNA-score	miRNA expression	Descriptive Explanatory	No validation	N/A	Expression in HR genes
Diossy [31]	WES-HRDetect	Mutational signatures LOH Indels	Predictive/ Descriptive	External	Sensitivity 76.6% AUC: 96%	LOH LST TAI *BRCA1/2* variants
Smyth [32]	Genomic LOH	Percentage of genomic LOH	Explanatory	No validation	N/A	Genomic LOH
Chen [33]	BRCA1-like classifier	Copy number	Predictive	External	AUC: 75%	MLPA assay (MRC-Holland)
Gulhan [34]	SigMA	Mutational signatures	Predictive Explanatory	Internal ^b^	Accuracy: 84% Sensitivity: 74%	Mutational Signature 3
Eeckhoutte [35]	ShallowHRD	Large-scale genomic alterations (LGA)	Predictive	External	Sensitivity: 87.5% Specificity: 90.5%	Variants or LOH in *BRCA1/2, RAD51C, PALB2* Methylation of *BRCA1* and *RAD51C*
Lips [36]	BRCA1-like digitalMLPA classifier BRCA2-like digitalMLPA classifier	Copy number	Predictive	External	BRCA1-like digitalMLPA classifier: Sensitivity: 93% Specificity: 90% Accuracy: 91% BRCA2-like digitalMLPA classifier: Sensitivity: 75% Specificity: 89% Accuracy: 82%	Algorithms developed by Joosse et al. [24] and Joosse et al. [22]
Nguyen [37]	CHORD	Single-base substitution Indels Structural variants	Predictive	External	Cohort 1: AUC: 98.7% Cohort 2: AUC: 99.5%	BRCA1/2 complete copy number loss LOH Germline or somatic variants in BRCA1/2
Barenboim [38]	DNA-methylation based RF classifier	Methylation copy number	Predictive	External	Sensitivity: 93% Specificity: 83% AUC: 87% Accuracy: 90%	Percent of genome change (PCG) score based on CNA, TAI, and LOH
Chen [39]	GSA	LOH LST TAI Ploidy	Predictive	Internal	Sensitivity: 95.2% (T) Specificity: 78.4% (T) AUC: 88.3 (T)	*BRCA1/2* variants LOH *BRCA1* methylation
Schouten [40]	Ovarian cancer BRCA1-like classifier Ovarian cancer BRCA2-like classifier	Copy number	Predictive	External	Ovarian cancer BRCA1-like classifier: Sensitivity: 96.2% Specificity: 40% Ovarian cancer BRCA2-like classifier: Sensitivity: 77% Specificity: 41%	*BRCA1/2* germline and somatic variants *BRCA1* methylation
Zhuang [41]	24-GPS	Gene expression	Predictive Explanatory	Internal	AUC: 98% (T)	Gene expression
Kang [42]	tHRD	Transcript usage	Predictive Explanatory	External	OC model: Accuracy: 72% BC model: Accuracy: 84%	LOH LST TAI Mutation Signature 3
Leibowitz [43]	HRD-DNA HRD-RNA	LOH Gene expression	Predictive Explanatory	External	HRD-DNA: Breast Sensitivity: 100% Specificity: 96.3% AUC: 100% F1: 98.3% HRD-DNA: Ovarian Sensitivity: 92.1% Specificity: 100% AUC: 99.3% F1: 95.9% HRD-RNA: prostate cancer Sensitivity: 85% Specificity: 98% AUC: 98% F1: 88% HRD-RNA: pancreatic cancer Sensitivity: 53% Specificity: 100% AUC: 98% F1: 69%	Biallelic loss of *BRCA 1/2*
Liao [44]	Transcriptomic HRD score	Gene expression	Predictive Explanatory	External	AUC: 79%	LOH LST TAI Deleterious *BRCA1/2* variants
Qu [45]	TD-score	Tandem duplications	Predictive Explanatory	Internal	AUC: 87% (T) Sensitivity: 88.2% (T) Specificity: 64.7% (T)	BRCA1-type HRD phenotype by CHORD [37]

Abbreviations: AUC: Area under the ROC curve; BC: Breast cancer; BRCA1: Breast cancer 1; BRCA2: Breast cancer 2; CHORD: Classifier of HOmologous Recombination Deficiency; GPS: Gene pairs (24-GPS); GSA: Genomic scar algorithm; HR: Homologous recombination; HRD: Homologous recombination deficiency; HRDS: Hypothesized HR-deficiency score; Indels: Insertion–deletion; LGA: Large-scale genomic alterations; LOH: Loss of heterozygosity; LST: Large-scale transitions; miRNA: MicroRNA; MLPA: Multiplex ligation-dependent probe amplification; N/A: Not available; NPV: Negative predictive value; OC: Ovarian cancer; PCG: Percent of genome changed; PPV: Positive predictive value; RF: Random forest; SCINS: Scores of chromosomal instability scarring; SigMA: Signature Multivariate Analysis; TAI: Telomeric allelic imbalance; TD: Tandem duplications; tHRD: Transcriptional HRD; WES: Whole-exome sequencing. a: Studies have been categorized into three different study types, based on the study objective and purpose being explanatory, predictive, and/or descriptive studies, as outlined in [25]. Explanatory studies cover studies such as causal inference, etiological, and association studies. Predictive studies cover studies such as prognostic, data mining, and machine learning studies, and descriptive studies aim to represent or describe data in a compact generalized way. b: The study used simulated panel data from down-sampled WGS data, which also acted as the gold standard.

## Supplementary Materials

The following supporting information can be downloaded at: https://www.mdpi.com/article/10.3390/cancers15235633/s1, Table S1: Search terms in Pubmed and Embase for the first and second search.

## List of Abbreviations

HRD	homologous recombination deficiency
PARPi	poly (ADP-ribose) polymerase inhibitors
HR	homolog recombination repair
BRCA1	breast cancer 1 gene
BRCA2	breast cancer 2 gene
LOH	loss of heterozygosity
LST	large-scale transition
TAI	telomeric allelic imbalance
PRISMA	Preferred Reported Items for Systematic Reviews and Meta-Analysis
ROC	receiver operating characteristic
PPV	positive predictive value
NPV	negative predictive value
TCGA	The Cancer Genome Atlas
GEO	Gene Expression Omnibus
SNP	single-nucleotide polymorphism
arrayCGH	comparative genomic hybridization array
FFPE	formalin fixed paraffin embedded
NGS	next-generation sequencing
WES	whole-exome sequencing
WGS	whole-genome sequencing
RNA-seq	RNA sequencing
SVM	support vector machine
LASSO	least absolute shrinkage and selection operator
CHORD	Classifier of HOmologous Recombination Deficiency
tHRD	transcriptional HRD
SigMA	Signature Multivariate Analysis
NMF	non-negative matrix factorization
LGA	large-scale genomic alterations
HRD score	combined homologous recombination deficiency score
GSA	genomic scar algorithm
SCINS	scores of chromosomal instability scarring
TD-score	tandem duplications score
HRDS	hypothesized HR-deficiency score (HRDS)
