# Supplementary material for: Homologous Recombination Deficiency Detection Algorithms: A Systematic Review
Source: Cancers (Basel). 2023 Nov 29;15(23):5633. doi: 10.3390/cancers15235633 (PMC10705160; doi:10.3390/cancers15235633)
Supplement: Supplementary file 1 [file cancers-15-05633-s001.zip › cancers-2688589-supplementary.pdf]

Supplementary Table S1: Search terms in Pubmed and Embase for the first and second search. The search strings were conducted by combining the search groups with ''AND'' and each search term within each search group with ''OR''. No filters were applied for the searches.

**Database** (**Search Number**)	**Search Groups**		
	**1. Homologous recombination deficiency**	**2. HRD Test**	**3. Data type/method**
**Pubmed (1)**	Homologous Recombination[MeSH Terms]	algorithms[MeSH Terms]	Databases, Genetic[MeSH terms]
	homologous recombination def[text word]	algorithm*[text word]	Sequence Analysis[MeSH Terms]
	homologous-recombination-def*[text word]	test[text word]	whole genome sequen*[text word]
	homologous recombination repair defiency*[text word]	tests[text word]	WGS[text word]
	homologous-recombination repair defiency*[text word]	score[text word]	whole-genome sequen*[text word]
	HR-defici*[text word]	scores[text word]	whole exome sequen*[text word]
	HR defici*[text word]	classifier[text word]	whole-exome sequen*[text word]
	HRD[text word]	script*[text word]	WES[text word]
	Homologous-recombination-repair[text word]	software[MeSH Terms]	next generation sequenc*[text word]
	Homologous recombination repair[text word]	software*[text word]	NGS[text word]
	HDR[text word]	tool[text word]	panel*[text word]
	HRR[text word]		PCAWG[text word]
	Homologous-recombination[text word]		Pancancer Analysis Whole Genomes[text word]
			Pan-Cancer Analysis Whole Genomes[text word]
			code[text word]
			High throughput sequencing[text word]
			OR International Cancer Genome Consortium[text word]
			ICGC[text word]

			array[text word]
			assay[text word]
			The cancer genome atlas[text word]
			TCGA[text word]
**Pubmed (2)**	Homologous Recombination[MeSH Terms]	algorithms[MeSH Terms]	Databases, Genetic[MeSH terms]
	homologous recombination def[text word]	algorithm*[text word]	Sequence Analysis[MeSH Terms]
	homologous-recombination-def*[text word]	test[text word]	whole genome sequen*[text word]
	homologous recombination repair defiency*[text word]	tests[text word]	WGS[text word]
	homologous-recombination repair defiency*[text word]	score[text word]	whole-genome sequen*[text word]
	HR-defici*[text word]	scores[text word]	whole exome sequen*[text word]
	HR defici*[text word]	classifier[text word]	whole-exome sequen*[text word]
	HRD[text word]	script*[text word]	WES[text word]
	Homologous-recombination-repair[text word]	software[MeSH Terms]	next generation sequenc*[text word]
	Homologous recombination repair[text word]	software*[text word]	NGS[text word]
	HDR[text word]	tool[text word]	panel*[text word]
	HRR[text word]	code[text word]	PCAWG[text word]
	Homologous-recombination[text word]		Pancancer Analysis Whole Genomes[text word]
	BRCAness[text word]		Pan-Cancer Analysis Whole Genomes[text word]
	BRCA-like[text word]		The cancer genome atlas[text word]
	BRCA1-like[text word]		High throughput sequencing[text word]
	BRCA2-like[text word]		International Cancer Genome Consortium[text word]
	BRCA* like[text word]		ICGC[text word]
	BRCAX like[text word]		array[text word]

	BRCA phenotype[text word]		assay[text word]
			TCGA[text word]
**Embase (1)**	'hr defici*':ti,ab,kw	software*:ti,ab,kw	panel*:ti,ab,kw
	hrr:ti,ab,kw	script*:ti,ab,kw	wes:ti,ab,kw
	'homologous recombination def':ti,ab,kw	algorithm*:ti,ab,kw	wgs:ti,ab,kw
	'homologous-recombination-def*':ti,ab,kw	tool:ti,ab,kw	'whole genome sequen*':ti,ab,kw
	'homologous recombination repair defiency*':ti,ab,kw	test:ti,ab,kw	'whole-genome sequen*':ti,ab,kw
	'homologous-recombination repair defiency*':ti,ab,kw	tests:ti,ab,kw	'whole exome sequen*':ti,ab,kw
	hrd:ti,ab,kw	score:ti,ab,kw	'whole-exome sequen*':ti,ab,kw
	hdr:ti,ab,kw	scores:ti,ab,kw	pcawg:ti,ab,kw
	'homologous-recombination-repair':ti,ab,kw	'algorithm'/exp	'pancancer analysis whole genomes':ti,ab,kw
	'homologous recombination repair':ti,ab,kw	classifier:ti,ab,kw	'pan-cancer analysis whole genomes':ti,ab,kw
	'homologous-recombination':ti,ab,kw	'genetic algorithm'/exp	icgc:ti,ab,kw
	'recombination repair'/exp	'software'/exp	'international cancer genome consortium':ti,ab,kw
	'homologous recombination'/exp		'high throughput sequen*':ti,ab,kw
			array:ti,ab,kw
			'genetic database'/exp
			assay:ti,ab,kw
			'the cancer genome atlas':ti,ab,kw
			tcga:ti,ab,kw
			'sequence analysis'/exp
			'dna sequencing'/exp
			'whole exome sequencing'/exp
			'high throughput sequencing'/exp
			ngs:ti,ab,kw
**Embase (2)**	'hr defici*':ti,ab,kw	software*:ti,ab,kw	panel*:ti,ab,kw
	hrr:ti,ab,kw	script*:ti,ab,kw	wes:ti,ab,kw
	'homologous recombination def':ti,ab,kw	algorithm*:ti,ab,kw	wgs:ti,ab,kw

	'homologous-recombination-def*':ti,ab,kw	tool:ti,ab,kw	'whole genome sequen*':ti,ab,kw
	'homologous recombination repair defiency*':ti,ab,kw	test:ti,ab,kw	'whole-genome sequen*':ti,ab,kw
	'homologous-recombination repair defiency*':ti,ab,kw	tests:ti,ab,kw	'whole exome sequen*':ti,ab,kw
	hrd:ti,ab,kw	score:ti,ab,kw	'whole-exome sequen*':ti,ab,kw
	hdr:ti,ab,kw	scores:ti,ab,kw	pcawg:ti,ab,kw
	'homologous-recombination-repair':ti,ab,kw	'algorithm'/exp	'pancancer analysis whole genomes':ti,ab,kw
	'homologous recombination repair':ti,ab,kw	classifier:ti,ab,kw	'pan-cancer analysis whole genomes':ti,ab,kw
	'homologous-recombination':ti,ab,kw	'genetic algorithm'/exp	icgc:ti,ab,kw
	BRCAness:ti,ab,kw	'software'/exp	'international cancer genome consortium':ti,ab,kw
	'BRCA-like':ti,ab,kw		'high throughput sequen*':ti,ab,kw
	'BRCA1-like':ti,ab,kw		array:ti,ab,kw
	'BRCA2-like':ti,ab,kw		'genetic database'/exp
	'BRCA phenotype':ti,ab,kw		assay:ti,ab,kw
	'BRCAX-like':ti,ab,kw		'the cancer genome atlas':ti,ab,kw
	'BRCA* like':ti,ab,kw		tcga:ti,ab,kw
	'recombination repair'/exp		'sequence analysis'/exp
	'homologous recombination'/exp		'dna sequencing'/exp
			'whole exome sequencing'/exp
			'high throughput sequencing'/exp
			ngs:ti,ab,kw
			'next generation sequenc*':ti,ab,kw
